# A 3DCNN-Based Knowledge Distillation Framework for Human Activity Recognition

**DOI:** 10.3390/jimaging9040082

**Published:** 2023-04-14

**Authors:** Hayat Ullah, Arslan Munir

**Affiliations:** Department of Computer Science, Kansas State University, Manhattan, KS 66506, USA; hayatu@ksu.edu

**Keywords:** deep neural networks, 3DCNN, deep learning, knowledge distillation, human action recognition

## Abstract

Human action recognition has been actively explored over the past two decades to further advancements in video analytics domain. Numerous research studies have been conducted to investigate the complex sequential patterns of human actions in video streams. In this paper, we propose a knowledge distillation framework, which distills spatio-temporal knowledge from a large teacher model to a lightweight student model using an offline knowledge distillation technique. The proposed offline knowledge distillation framework takes two models: a large pre-trained 3DCNN (three-dimensional convolutional neural network) teacher model and a lightweight 3DCNN student model (i.e., the teacher model is pre-trained on the same dataset on which the student model is to be trained on). During offline knowledge distillation training, the distillation algorithm trains only the student model to help enable the student model to achieve the same level of prediction accuracy as the teacher model. To evaluate the performance of the proposed method, we conduct extensive experiments on four benchmark human action datasets. The obtained quantitative results verify the efficiency and robustness of the proposed method over the state-of-the-art human action recognition methods by obtaining up to 35% improvement in accuracy over existing methods. Furthermore, we evaluate the inference time of the proposed method and compare the obtained results with the inference time of the state-of-the-art methods. Experimental results reveal that the proposed method attains an improvement of up to 50× in terms of frames per seconds (FPS) over the state-of-the-art methods. The short inference time and high accuracy make our proposed framework suitable for human activity recognition in real-time applications.

## 1. Introduction

Deep neural networks (DNNs) have immensely improved over the years and have shown remarkable success in various fields such as computer vision [1], natural language processing [2], speech recognition [3], and other scientific research domains. The efficiency of DNNs generally depends on the design of network architecture (depth and formation of network layers) for the task under consideration. For the tasks such as object recognition [4] and video analytics [5], mostly the DNNs are over-parameterized to ensure generalization and capturing of the complex hidden patterns in the data. Such over-parameterized and cumbersome models are very deep and computationally complex, which require substantial amount of computational resources for training and are not applicable for real-time environments. To achieve computationally efficient yet robust models with reasonable inference speed, computer vision researchers have been actively working to exploit the effectiveness of large trained models to acquire computationally efficient light-weight models, which can be used for real-time applications. Besides the model complexity, the other key factor which plays an important role in the performance of DNNs is data. For problems such as video analytics (human action recognition, person identification, and object tracking), the DNNs models highly require large amount of labeled data for training. However, in many cases it is difficult to have enough amount of highly accurate labeled training data for the task under the consideration.

To alleviate the challenge of a large labeled dataset requirement, computer vision researchers have introduced several solutions including semi-supervised learning [6], self-supervised-learning [7], and knowledge-distillation [8] that reduce the burden of labeling large datasets by training DNNs models on partially labeled datasets. In semi-supervised learning paradigm, a DNN model is trained on a data having a small portion of labeled and a large portion of unlabeled data. First, the DNNs model is trained on a small portion of labeled data, then later the trained model generates the pseduo-labels with a certain confidence level (prediction of the trained model). The generated pseduo-labels are then added to the labeled pool of data based on the corresponding confidence scores. After labeling the entire dataset, the pre-trained model is trained again on the new labeled dataset to improve performance. On the other hand, in self-supervised learning paradigm, a DNN model is trained on an artificially constructed labeled data (constructed using transformations such as rotation, flipping, and color change, etc.) where the model learns and supervises itself from the knowledge of predefined transformations. The knowledge distillation approach is a special variant of self-supervised learning which offers both model compression and knowledge transfer. The paradigm knowledge distillation consists of two network architectures namely teacher and student, where the student learns from the teacher during training by minimizing the mutual loss known as the *distillation loss*.

For the first time, the concept of knowledge distillation was introduced by Hinton et al. [8], where they investigated the characteristics of knowledge distillation in terms of model compression and knowledge transfer from the source model to the target model. In knowledge distillation, the source model refers to the *teacher model* and the target model refers to the *student model*, the pair of teacher-student model learns together during training and improves the task-specific performance by minimizing the mutual loss (distillation loss). For model compression, the student model is the smaller version of the teacher model, where the smaller student model tries to improve itself during training by capturing the same level of patterns from the data and the same level of prediction accuracy as the teacher model. After the training phase, the distilled student model performs same level of predictions as the teacher model despite having comparatively less parameters. Generally, knowledge from the teacher model can be transferred to the student model at different levels, that include response-based, features-based, and relational-based knowledge distillation. In response-based knowledge distillation, the student model learns the class distribution predicted by the teacher model (soft labels or probabilities) by minimizing the loss between the logits (i.e., vector of raw and unnormalized predictions generated by the last linear layer of a neural network before it is passed to a softmax or other such normalization) of the student and the teacher model. The features-based knowledge distillation transfers the features representation level knowledge from the teacher to the student model by minimizing the features-level difference between the teacher and the student model. Generally, the teacher model has a deeper architecture and has better representation of features than the student model. Therefore, the feature-based knowledge distillation technique uses the intermediate layers of the teacher model to train the student model to extract the same level of features representation as the teacher model. Lastly, the relation-based knowledge distillation uses the relationship between the feature maps of teacher model to enhance the relationship between the feature maps of student model by minimizing the mutual distillation loss.

This paper mainly focuses on the concept of knowledge distillation for the task of human action recognition in videos. Considering the time-series nature of video data and the complexity of recognizing human actions in time series, where the position and movement of human and other objects vary across the video frames, we propose a 3D convolutional neural network (3DCNN)-driven knowledge distillation framework that consists of two 3DCNN networks namely 3DCNN teacher and 3DCNN student. For efficient knowledge transfer, we propose an offline knowledge distillation strategy, where the 3DCNN teacher model is pre-trained and the 3DCNN student model is untrained. It is worth mentioning here that, for knowledge distillation both models must share the same dataset, therefore, we first train the 3DCNN teacher model on a dataset and then use the knowledge distillation to transfer the knowledge from the trained 3DCNN teacher model to 3DCNN student model over the same dataset. To summarize, the main contributions of this work are as follows:

1. We propose two 3DCNN models (i.e., a teacher and a student model) to extract spatial and temporal features from video sequences. The network architecture of the proposed teacher model is based on the C3D [9] model having a large parameter space whereas the proposed student model is a shallow version of the teacher model having 10× less parameters then the teacher model.
2. We propose a response-based offline knowledge distillation framework for human action recognition, utilizing the knowledge of a large pre-trained teacher model to efficiently train a student model. During training, the offline knowledge distillation algorithm helps the lightweight student model to attain the same level of prediction accuracy as the large pre-trained teacher model by minimizing the distillation loss.
3. By conducting extensive experiments, we obtain a computationally efficient knowledge distillation-driven 3DCNN student model, which has 10× less parameters than the teacher model. Further, the developed 3DCNN student model has a storage requirement of 28 MB, which is 11× less than the size of the 3DCNN teacher model having the model size of 321 MB, yet providing the same level of prediction accuracy as the teacher model.
4. We evaluate and compare the run time efficiency of the proposed method with the state-of-the-art human action recognition methods. Experimental results demonstrate that the proposed method attains an improvement of up to 37× in terms of seconds per frame (SPF) metric and up to 50× in terms of frames per second (FPS) metric over the state-of-the-art methods.

The rest of this paper is organized as follows. Section 2 presents a brief overview of the related works. The proposed framework along with its components is discussed in detail in Section 3. Section 4 presents an extensive experimental evaluation of the proposed framework and detailed comparisons with the state-of-the-art human action recognition methods. Finally, Section 5 concludes this paper with possible future research directions in human action recognition domain.

## 2. Related Works

The topic of human activity recognition has been actively studied over the last ten years, where numerous advanced methods have been presented to efficiently address the human action recognition problem. The existing literature of vision-based human action recognition reports several spatial-temporal approaches. Broadly, the existing approaches can be categorized into two-stream 2DCNN-based methods [10,11,12,13,14], recurrent neural network (RNN)-based methods [15,16,17,18,19], and 3DCNN-based methods [9,20,21,22,23,24]. The two-stream 2DCNN-based methods contain two distinct 2DCNN architectures that take same input (series of RGB (red, green, and blue) frames) and extract different level of features (such as discriminative spatial and optical flow features, etc.), and then the extracted features are combined using specific fusion techniques. For instance, Karpathy et al. [10] proposed a two-stream 2DCNN approach, where they fed different resolution inputs (low-resolution and high-resolution RGB video frames) to two different CNN networks to expedite the computation process. Later, they fused the extracted features from the two different networks using various fusion strategies to model the spatio-temporal dynamics of videos. Cheron et al. [11] utilized the joints of human body for cropping the RGB and optical flow frames based on human positions, the cropped RGB and optical flow frames are then fed to two different CNN networks for feature extraction. The extracted features from the two distinct CNN networks were then forwarded to a support vector machine classifier for final human action recognition. Wang et al. [12] proposed a two-stream 2DCNN network together with video frames segmentation strategy. They first segmented a given video in three different segments and then fed each to two-stream 2DCNN network for segment classification. Later, they combined the classification scores of the three segments using average pooling to obtain video-level classification.

In the work presented in [13], Girdhar et al. used two different levels of representation of input video frames that included sampled visual appearance frames and motion-specific frames. They fed visual appearance and motion frames to a two-stream 2DCNN network. The extracted features from the two different networks were then aggregated using a vocabulary (called action words) for video-level representation and classification task. In another work, Zhang et al. [14] proposed a multi-task framework for human action recognition in low-resolution videos. For super-resolution task, they proposed two video super-resolution methods which enhanced the visual quality of the low-resolution videos. The reconstructed high-resolution videos were then fed to two-stream spatio-temporal networks for video-level human action classification. In short, the two-stream 2DCNN methods extract different types of features (i.e., spatial and temporal) using two distinct network architectures from the same input video data. Generally, the extracted features of different types are then fused as a combined feature representation for efficient recognition of human actions in videos. However, these types of methods are still lacking the efficiency to cope with long-term sequential modeling and action recognition tasks.

On the other hand, RNN-based methods reported in the literature typically use single 2DCNN for spatial features extraction followed by the RNN network for temporal patterns learning. The literature reports that RNNs perform comparatively better than CNNs used for temporal features extraction due to their recurrent nature which can efficiently learn hidden temporal patterns. However, RNNs are shown vulnerable to vanishing gradient problem while dealing with long-term temporal patterns. To cope with this challenge, computer vision researchers have introduced gated RNN such as long short-term memory (LSTM) [25] and gated recurrent unit (GRU) [26] that are able to capture long-term temporal patterns or relation between the temporal patterns. Several existing human action recognition methods have adopted LSTM for efficient temporal modeling of human actions in video sequences. For example, Donahue et al. [15] proposed a long-term recurrent convolutional network called LRCN, which consisted of CNN and LSTM networks. The CNN network is used for frame-level spatial features extraction, where the LSTM network was used to capture temporal patterns in the extracted CNN features for video level action recognition task. Srivastava et al. [16] presented an unsupervised LSTM encoder and decoder networks for human action recognition task. The LSTM encoder network transformed the input video to a fixed length of spatio-temporal representation. The decoder network was then used for reconstruction of videos from latent spatio-temporal representation and prediction of human actions in reconstructed videos.

In the work proposed in [17], Sharma et al. used multi-layer LSTM network for recursive computation of attention maps and prediction of subsequent frames. They fed the RGB features of video frames to a multi-layer LSTM, which computed weighted attention maps by performing recursive operations. They claimed that the weighted attention maps helped the network to focus on salient features and perform better predictions. Li et al. [18] presented a method called Video-LSTM. Their proposed method incorporated spatial CNN features and motion-specific attention maps. They utilized the soft-attention of their proposed LSTM network to efficiently capture spatio-temporal patterns for human action recognition task. In another work, Sudhakaran et al. [19] presented an LSTM-based approach for action recognition task. They introduced a special variant of recurrent unit with built-in spatial attention mechanism to localize the salient information in video sequences. The salient spatial information was then fed to the LSTM network for the recognition of human actions in videos.

Generally, the RNN-based methods are coupled with 2DCNN architectures, where 2DCNN transforms the input videos to latent represention and the RNNs operate on the extracted CNN features. Although, these methods have shown reasonable performance for human action recognition task, they can be replaced with unified network architectures that offers the same level of recognition performance.

Beside the two-stream 2DCNN-based and RNN-based architectures, several studies have extended the 2DCNNs to 3DCNNs that have the ability to capture both spatial and temporal features from the input RGB video frames. For instance, Tran et al. [9] proposed a 3DCNN model known as C3D to capture the spatio-temporal features from input raw videos in end-to-end fashion. They trained and evaluated their C3D network on small video clips (each clip has 16 frames) instead of full videos. Using small video clips instead of full videos, however, can miss the long-term spatio-temporal dependencies in the video sequences. Therefore, numerous methods are proposed to cope with the long-term spatio-temporal dependencies in videos. To address the issue of long-term dependency in videos, Diba et al. [20] presented an extended version of DensNet [27] known as temporal 3DCNN (T3D) by replacing the 2D spatial kernels and 2D pooling kernels with 3D spatial kernels and 3D pooling kernels. They claimed that their developed T3D model could effectively capture both spatial appearance and temporal flow both in short-term and long-term temporal video sequences. Varol et al. [21] introduced a long-term temporal convolution (LTC) network. They increased the temporal depth of their 3DCNN layers and reduced the spatial resolution of feature maps to efficiently capture the long-term temporal patterns in video sequences. In another study, Diba et al. [22] introduced a special variant of ResNet architecture by replacing the 2DCNN blocks with 3DCNN blocks. They claimed that their developed architecture could model the channel-wise correlations of a 3DCNN with respect to the spatio-temporal features. Hussein et al. [23] introduced a multi-scale temporal-specific 3DCNN network called Timeception. Their proposed method was developed to deal with the extreme variations in temporal dimension by tolerating various temporal extents for recognizing long and complex actions. To capture long-term dependencies in video sequences, Li et al. [24] introduced a 3DCNN network called channel independent directional convolution (CIDC). Authors claimed that their proposed CIDC model could be used together with I3D model [28] to efficiently capture long-term temporal dependencies of full-length videos.

In general, the existing two-stream 2DCNN, RNN-based, and 3DCNN-based methods are very efficient in terms of modeling discriminative salient features from both spatial and temporal dimensions features. However, these methods are mostly based on computationally complex architectures (having tens of millions of parameters) which require gigantic amount of training data, large storage, and high computational power. To alleviate the computational burden of the aforementioned deep learning methods for human activity recognition task, numerous knowledge distillation-based methods are presented that compress the model size by transferring the knowledge from a large model (teacher) to a small model (student). For instance, Hao and Zhang [29] proposed spatio-temporal distilled dense-connectivity network (STDDCN) for human action recognition in video streams. Their proposed method explored reciprocity between motion streams and appearance with different hierarchies. Authors claimed that the knowledge distillation and fusion between the two streams allow both streams to learn resilient features at high level layers. Liu et al. [30] proposed an attention distillation-based method for learning video representation. They explored different attention choices and distilled motion-aware knowledge from the source pre-trained optical flow model to their proposed RGB model. To learn video representation in extremely low-resolution videos, Purwanto et al. [31] presented a spatio-temporal two-stream network with a self-attention mechanism. They first used super-resolution to enhance the visual quality of the low-resolution videos for training their proposed network. Further, they utilized knowledge distillation mechanism to transfer the spatio-temporal knowledge from a teacher (trained on high-resolution data) to a student model (to be trained on low-resolution data). In the work presented in [32], Stroud et al. first investigated motion representations in spatial stream and demonstrated significant room for further improvements in the performance. Second, they demonstrated that motion representations could be improved using knowledge distillation, that is, by distilling the knowledge from the temporal stream to the spatial stream. They then effectively fused both the streams into a single stream.

Continuing research efforts in the same directions, Vu et al. [33] proposed a self-knowledge distillation method based on siamese representation learning. We note that a siamese representation learning leverages a *siamese neural network*, which is sometimes also referred to as a twin neural network. A siamese neural network is an artificial neural network that comprises of two or more identical subnetworks, that is, the subnetworks have the same configuration with the same parameters and weights. Vu et al. [33] method minimized the difference between the two representation vectors (generated from a siamese neural network) of video frames captured from different views. Their proposed method utilized both similarity of representation vectors and soft label distillation for learning efficient video representation and human action prediction. In another work, Vu et al. [34] proposed a self-knowledge distillation approach known as Teaching Yourself for action recognition in video stream. They leveraged self-knowledge distillation mechanism to train a student model progressively by transferring its own knowledge without using a pre-trained teacher model. In their proposed training strategy, the network under training updated itself using the best past model called the preceding model, which was developed to guide the training process and update the present model. Zhou et al. [35] presented a novel transfer learning method which combined self-distillation in fine-tuning to preserve the knowledge of a pre-trained teacher model learned from a large-scale video dataset. Further, they fixed the encoder as a teacher model in the last epoch to guide the training of the encoder from the current epoch in transfer learning phase. In the work presented in [36], Vu et al. proposed an unsupervised distillation learning framework called (2 + 1)D Distilled ShuffleNet to train a lightweight model for human action recognition task. By leveraging the distillation technique, they developed (2+1)D Distilled ShuffleNet as an unsupervised approach, which did not require labeled data for training. Further, they evaluated the performance of their method by distilling the knowledge from two different teachers that included a 2DCNN teacher and a 3DCNN teacher. Tran et al. [37] presented a novel framework that incorporated progressive knowledge distillation for early human action recognition. They used two RNN networks, i.e., a teacher and a student, where they distilled the knowledge from the teacher model to the student model using self-distillation approach. Shalmani et al. [38] proposed a knowledge distillation approach called confidence distillation framework, which guided a student model regarding how to choose an appropriate video for the teacher to predict.

To overcome the limitations of training a cumbersome 3DCNN model on large video data, in this work, we propose a knowledge distillation-driven lightweight 3DCNN architecture having a total of eight convolution layers. The proposed offline knowledge distillation algorithm facilitates both knowledge transfer and model compression in a single unified framework, which allows us to transfer response-based knowledge from a large pre-trained 3DCNN teacher model to a lightweight 3DCNN student model. Thus, the proposed 3DCNN architecture (i.e., the student model) provides the same recognition performance (i.e., prediction accuracy) as the 3DCNN teacher model, while having a far less network complexity than the 3DCNN teacher model.

## 3. The Proposed Spatio-Temporal Knowledge Distillation Framework

This section provides the detailed architecture and working of the proposed spatio-temporal knowledge distillation 3DCNN framework ant its major components. The proposed spatio-temporal knowledge distillation framework is based on three major components that include teacher-student 3DCNN architectures, offline knowledge distillation paradigm, and workflow of spatio-temporal knowledge distillation process. The first core component of the proposed framework is the pair of teacher-student 3DCNN networks, which are developed to capture spatio-temporal features from the input videos frames. The second major component of the proposed framework is the offline knowledge distillation paradigm, which uses a pre-trained model as a teacher or a source model and transfer the knowledge to a student or a target model. In this work, we have used offline knowledge distillation, where we use a pre-trained 3DCNN as a teacher model and an untrained 3DCNN with reduced computational complexity as a student model. The third and last component of the proposed framework is the spatio-temporal knowledge distillation process, which transfers both spatial and temporal knowledge from the 3DCNN teacher to the 3DCNN student model. Each major component is discussed in detail in subsequent subsections.

### 3.1. Proposed Teacher-Student 3DCNN Architectures

**Figure 1:**
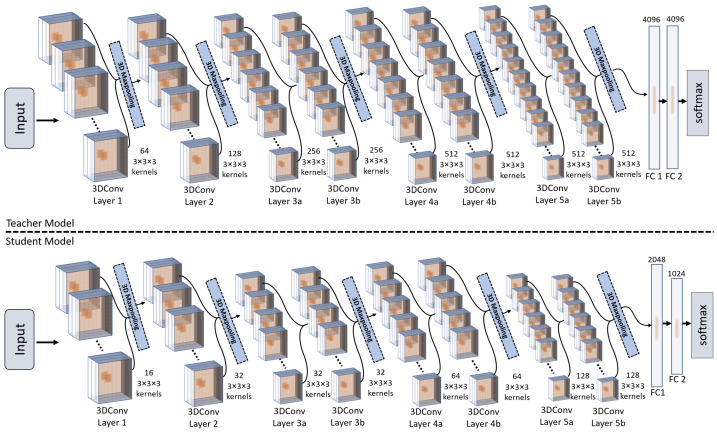
The visual overview of the proposed 3DCNN Teacher and 3DCNN Student network architectures.

**Figure 2:**
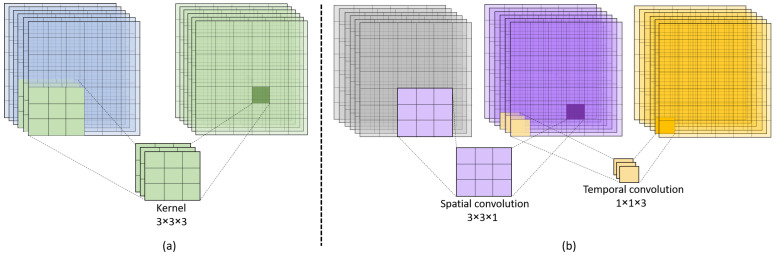
The visual overview of 3D kernel operation. (**a**) Combined 3D Convolution kernel operation on stack of frames, (**b**) (2D+1D) kernels separate operations on input frames for spatio-temporal features extraction.

In this section, we present the architectural details of the proposed teacher-student 3DCNNs. The architectural design of the proposed teacher-student models is inspired by the C3D model presented in [9]. Originally, the C3D model [9] is trained on the Sports-1M dataset, which has around 79.9 million trainable parameters. Considering the depth of the original network and its feature extraction efficiency, we have used the same architecture for our teacher network and have used the smaller version (having lesser number of training parameters) of C3D as a student model by reducing the number of 3D kernels per layer. The architectural details of the developed teacher and student 3DCNNs are depicted in Figure 1. As it can be noticed from the given figure that, the teacher network has a large number of 3D convolution filters per layer as compared to the student model, and therefore such a layer-wise depth of the teacher model leads to a significantly high computational complexity in terms of trainable parameters of the network. The convolution layers of 3DCNN model have the ability to learn both spatial and temporal features using a series of 3D kernels. Typically, the convolution filters of 3DCNN have three dimensions (H,W,D), where the first two dimensions *H* and *W* operate on spatial resolution of the input frames and the third dimension *D* performs temporal feature extraction from a series of frames as shown in Figure 2a,b. More precisely, the 3D convolution operation can be consider as (2D+1D) convolution, where the 2D convolution operates on the spatial dimensions of the image by applying (3×3×1) kernels for capturing spatial features and the 1D convolution operates on the temporal dimensions of input frames by applying (1×1×3) kernels for learning temporal features as depicted in Figure 2b. Similarly, 3D pooling layer down-samples the spatial dimension of the input feature maps across each channel and dimension by shifting pooling kernels with specified stride. For padding, we use zero-padding of 1, which adds pixels with value zero at boundaries of the feature maps at each convolution layer with thickness of padding equal to one pixel. Further, the design of our developed teacher and student 3DCNN models are based on the motivation of model compression, where the small student model (having 7 millions trainable parameters) learns from the large teacher model (having 78 million trainable parameters) to perform at the same level of action prediction accuracy while having approximately 11× less trainable parameters than that of teacher model. Therefore, the scope of this work includes both model compression and spatio-temporal knowledge distillation. The outcome of the proposed method will be a computationally efficient yet robust 3DCNN student model, which inherits the prediction performance from the teacher model while having significantly lower computational complexity. Having such a computationally efficient yet robust model, will enable us to use it for resource-constrained edge devices as well as for real-time applications [39]. More precisely, the detailed insights of the proposed teacher and student 3DCNN network architectures are presented in Table 1 and Table 2, respectively. As it can be seen in Table 1 and Table 2 that the number of layers in both the teacher and the student network are the same, however the number of kernels per layer in the student model is less than that of the teacher model. Further, the padding and the stride per convolution and the pooling layers are same in both the networks (i.e., the teacher and the student 3DCNNs). Furthermore, the size of the fully connected layers (having latent representation of frames) of the 3DCNN student model is smaller than that of the 3DCNN teacher model.

### 3.2. Offline Knowledge Distillation Paradigm

**Figure 3:**
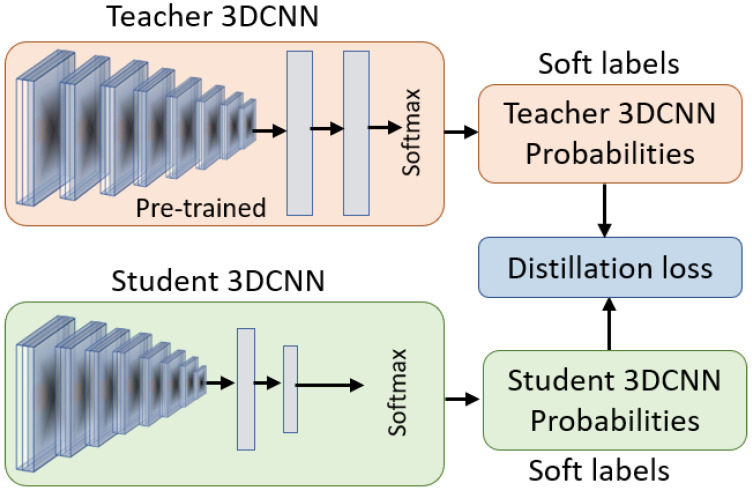
The visual overview of the proposed offline knowledge distillation approach, transferring knowledge from the pre-trained teacher to the student model.

Generally, in terms of knowledge transfer, the knowledge distillation paradigm can be divided into two categories: *online knowledge distillation* and *offline knowledge distillation*. In the online knowledge distillation, the teacher and the student model train and update simultaneously in the end-to-end training process. Here, both the teacher and the student model(s) learn collaboratively from each other on the same input data in a peer learning fashion. In this knowledge distillation paradigm, the teacher and the student(s) learn from the predictions of each other to improve their prediction accuracy. However, the predictions of the teacher and the student(s) can vary at any point during the training phase, where the output of the teacher and the student models can conflict with each other and even with the ground truth. In cases where the predictions of the teacher and the student vary over the training phase, the online knowledge distillation can greatly harm the performance of the distilled student model. On the other hand, in the offline knowledge distillation paradigm, the student model learns from a single pre-trained teacher model as shown in Figure 3.

The motivation behind using the offline knowledge distillation is that the knowledge from the cumbersome pre-trained teacher model can greatly help the student model to perform the predictions with a similar accuracy. During the training phase, the teacher model normally starts converging in the very early epochs due its deep architecture, whereas the student model takes time to land on the global minima which ensures the convergence of the model. As a result, the performance of the teacher model (in terms of predictions) enforce the student model to get better in predictions over the training period. Typically, at the end of each epoch in the forward pass, the distillation loss (cross-entropy loss) computes the difference between the teacher’s and the student’s predictions, which helps the student to adjust its weights in the backward pass and improve its predictions accuracy performance. Thus, using the offline knowledge distillation approach enables us to obtain a computationally efficient yet a robust model which offers the same level of performance as the teacher model.

### 3.3. Spatio-Temporal Knowledge Distillation from Teacher to Student Model

**Figure 4:**
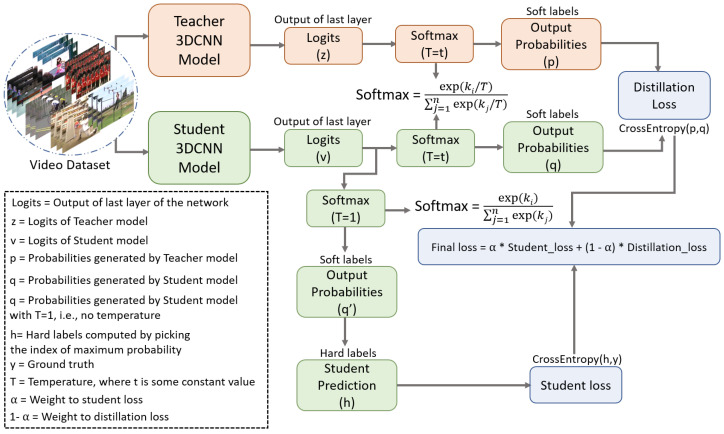
The workflow diagram of the proposed spatio-temporal knowledge distillation method using offline distillation approach.

This section presents the working procedure of the proposed spatio-temporal knowledge distillation framework. As we discussed in the previous section that the proposed framework is developed to transfer the knowledge from the cumbersome pre-trained teacher model to the lightweight student model using the offline knowledge distillation approach. To use a pre-trained model for the offline knowledge distillation, we first train the 3DCNN teacher model and then use the trained 3DCNN teacher model in an offline knowledge distillation training module, which supervises the student model in training based on its predictions and the student’s predictions. In the offline knowledge training, the teacher model only performs predictions, whereas the student model trains and updates its weights based on the difference between the predictions of the teacher and the student model, which helps the student model to improve its prediction accuracy based on the teacher’s predictions. The workflow diagram of the proposed sptio-temporal knowledge distillation method is depicted in Figure 4. From Figure 4, it can be perceived that the proposed method transfers the response-based (i.e., predictions) knowledge from the pre-trained 3DCNN teacher model to the 3DCNN student model by computing the distillation loss between the teacher’s predictions (*p*) and the student’s predictions (*q*). The computed distillation loss enforces the student model to adjust its weight to minimize the distillation loss. It is worth mentioning here that for the distillation loss as given in Equation (1), both the teacher and student models produce the probabilities (soft labels) using normalized or soften softmax function as given in Equation (2).

(1) Distillation_Loss=CrossEntropy(p,q),

(2) $$Normalized\_Softmax=\frac{exp(k_{i}/T)}{\sum_{j=1}^{n}exp\left(k_{j}/T\right)},$$

Here the terms *p* and *q* in Equation (1) represent the probability vectors produced by the teacher and the student model, respectively. The distillation loss in Equation (1) computes the difference between the predicted probabilities *p* and *q* and provide a scalar loss value. The term $k_{i}$ in Equation (2) represents a single instance of the logits (the values of the last fully connected layer) and the variable *T* denotes a constant temperature value. Here, the role of temperature *T* value in the normalized softmax function is to produce normalized or smooth probability vectors. Normally, the probability vector produced by the standard softmax function has non-uniform distribution of probability values. Furthermore, the probability vectors of two different models for the same class can vary to a high extent, which makes it infeasible to compute the generalized loss value. Therefore, each value of logits vector in the softmax function is divided by *T* to provide the uniformly distributed probability vectors for both teacher and student model as shown in Figure 4. A normalized softmax function with different temperature *T* values will result in different probability vectors (soft labels). Therefore, we have considered different temperature *T* values in our experiments to observe its impact on the knowledge distillation performance. Thus, in our proposed framework, *T* is a hyperparameter which can be tuned to provide the best prediction accuracy. The student loss as given in Equation (3) computes the difference between the student’s prediction and the ground truth based on which the student model then generates the output class probabilities using standard softmax function as given in Equation (4). The reason for using standard softmax function instead of the normalized softmax function is that the student loss computes the difference between the predictions made by the student and the ground truth.

(3) Student_Loss=CrossEntropy(h,y),

(4) $$Softmax=\frac{exp(k_{i})}{\sum_{j=1}^{n}exp\left(k_{j}\right)},$$

Here in Equation (3), the variable *h* represents the final predictions for a specific class (derived from the student probability vector) and the variable *y* denotes the ground truth. The final loss function is the weighted sum of the distillation loss and the student loss as given in Equation (5)

(5) Final_Loss=α×Student_Loss+(1−α)×Distillation_Loss,

where Student_Loss and Distillation_Loss denote the student loss and the mutual distillation loss of the teacher and the student model, respectively, and the variable α is the weight factor of the final loss that defines contribution of the student and the distillation loss in the final loss.

## 4. Experimental Results and Discussion

In this section, we present the comprehensive experimental evaluation of our proposed framework on different datasets for human action recognition task. First, we briefly describe the implementation details and tools we have used to conduct our experiments. Next, the datasets used in this research are briefly discussed, followed by the comparative analysis of the results we have obtained with different settings of our proposed framework. Finally, we present the comparative analysis of our proposed framework with the state-of-the-art human action recognition methods.

### 4.1. Implementation Details and Tools

To implement our proposed framework, we have used Python language 3 and TensorFlow 2.0 Framework on a computing system with Intel(R) Xeon(R) CPU E5-2640 having processor frequency of 2.50 GHz and 32 GB of dedicated main memory (i.e., RAM). The utilized computing system is equipped with two Tesla T4 GPUs having compute capabilities of 7.5 with Nvidia CUDA library version 11.0. For training and validation, we have divided the datasets into subsets of 70% and 20% data, where the 70% data is used for training and 20% is used for validation. The remaining 10% data is used for testing the performance of the trained models. We set the epoch value 100 to train the models for 100 epochs with different different settings (i.e., different teacher and temperature values). To adjust the model weights, we have used Adam optimizer with a static learning rate of 0.0001 and categorical cross entropy loss. To learn spatio-temporal features, we set the input sequence length to 16 frames for the proposed 3DCNN framework. The 3DCNN learns the spatio-temporal features by sliding 3D kernels on the input sequence of 16 frames. Moreover, to analyze the performance of the proposed framework in a more generalized way, we have used two different performance evaluation metrics that include accuracy and run time analysis assessment. The accuracy metric is used to evaluate video-level prediction performance of the proposed frameworks, where the run time analysis assessment metric is used to analyze the run time of the proposed framework.

### 4.2. Datasets

To evaluate the effectiveness of the proposed methods, extensive experiments have been conducted on different human actions datasets that include UCF11 [40], HMDB51 [41], UCF50 [42], and UCF101 [43] datasets. Each dataset contains numerous videos having different actions, viewpoints, and motion variations. For instance, the UCF11 dataset [40] consists of 1640 video clips categorized into 11 distinct human actions, where each class contains multiple videos of the same action having different viewpoint, number of humans, and motion variation. HMDB51 dataset [41] is one of the challenging datasets, containing 6849 video clips of human actions and categorized into 51 distinct human action classes. The dataset is annotated with a text label for each video clip and meta-label to provide the properties of video clip such as viewpoint, frames per second, camera motion, and number of individuals involved in action. The UCF50 dataset [42] is a widely used human actions benchmark dataset, containing 6676 video clips of human actions categorized into 50 distinct human action classes. Finally, the UCF101 dataset [43] is the extended version of UCF50 dataset [42], containing 13320 video clips categorized into 101 different actions classes. Each class has approximately 100 to 200 video clips, where each video has a duration of 2 to 3 s with a frame rate of 25 FPS.

### 4.3. Assessment of the Baseline Results

**Figure 5:**
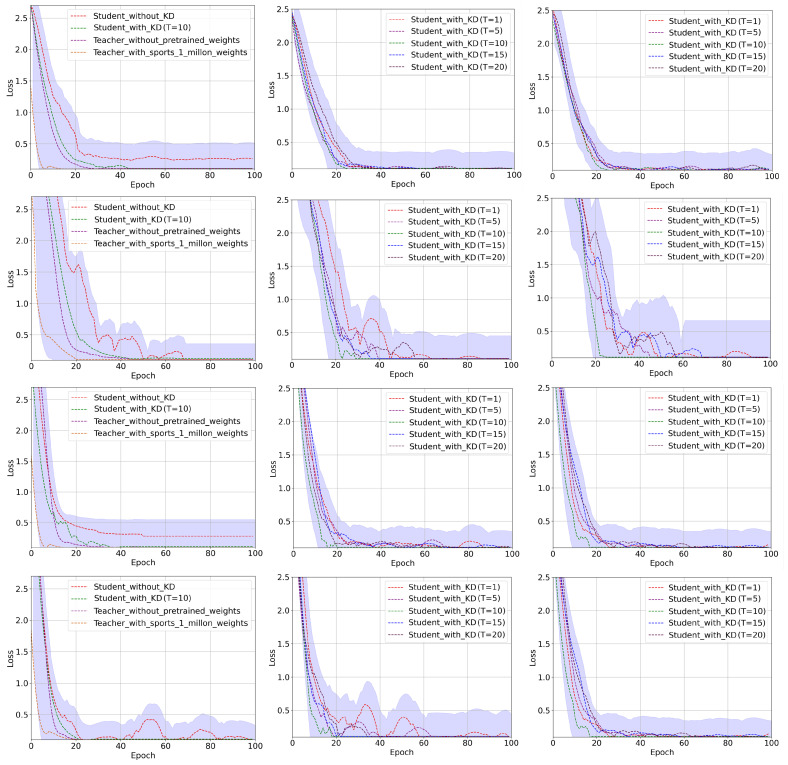
The visual overview of training histories for teacher and student models trained with different settings. The first, second, third, and fourth row represent the average training loss of teacher and student model with different settings over UCF11, HMDB51, UCF50, and UCF101 datasets, respectively.

**Figure 6:**
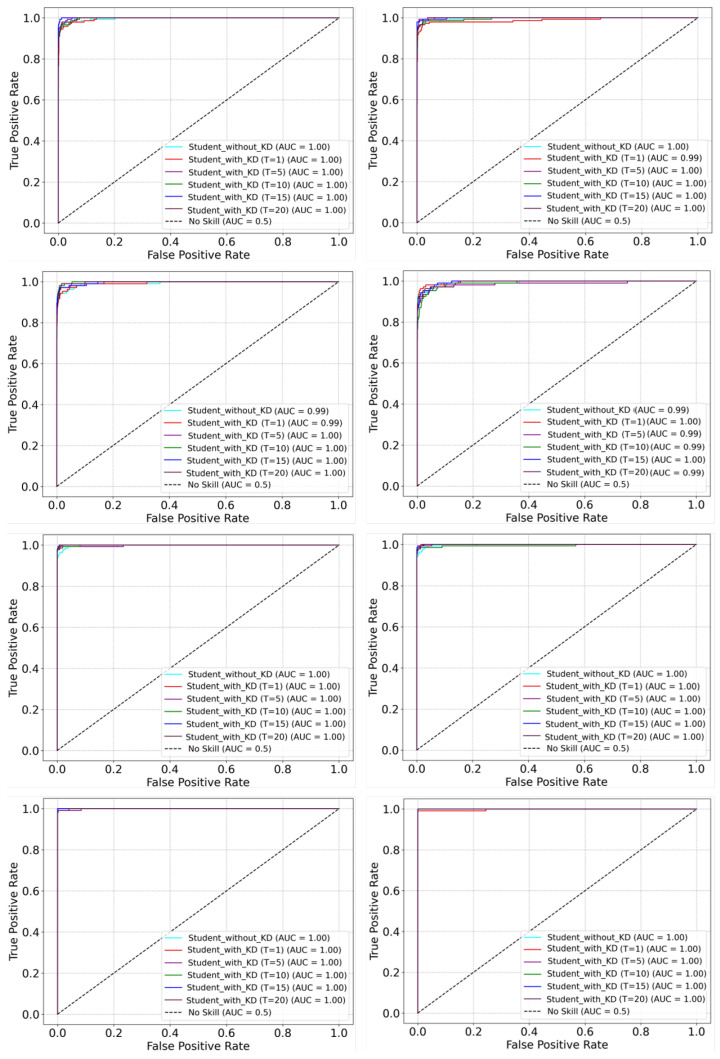
The receiver operating characteristics (ROC) curve graphs of our proposed 3DCNN student model for UCF11, HMDB51, UCF50, and UCF101 datasets. First column represents the ROC curve graphs of the proposed 3DCNN student model distilled with the the TFS teacher model, whereas the second column represents the ROC curve graphs of the proposed 3DCNN student model distilled with the TUTL teacher model.

In this research, we have explored the spatio-temporal knowledge distillation from a pre-trained 3DCNN teacher model to a 3DCNN student model with different settings. We have also evaluated the performance of a light-weight 3DCNN model (student) with and without knowledge distillation. To obtain a well-generalized yet an efficient model across each dataset, we have conducted extensive experiments with different settings in our knowledge distillation framework. As our proposed method is based on the offline knowledge distillation approach, which transfers spatio-temporal knowledge from a large 3DCNN teacher model to a lightweight 3DCNN student model. Therefore, first we have trained the 3DCNN teacher model and later we have used the pre-trained 3DCNN model in knowledge distillation training. It is worth mentioning here that we have trained the 3DCNN teacher model with two different settings that include training from the scratch, which we refer to as TFS, and the training using transfer learning from [9] (using its pre-trained weights), which we refer to as TUTL. In the first setting, we have trained the 3DCNN teacher model TFS from scratch on each dataset and then used it for knowledge distillation. In the second setting, we have trained the 3DCNN teacher model TUTL with pre-trained weights from [9] using transfer learning technique. We have assessed the performance of knowledge distillation with the two different pre-trained teacher models. Further, we also have investigated the effect the impact of temperature values (a hyperparameter in our knowledge distillation framework as shown in Figure 4) on knowledge distillation, where we have examined the spatio-temporal knowledge distillation with different temperature values (i.e., T = 1, 5, 10, 15, 20) for both the pre-trained teacher models. The training histories for a set of conducted trainings are depicted in Figure 5. For instance, in Figure 5, the left-most plots represent the average training losses for the 3DCNN teacher TFS, for the 3DCNN teacher TUTL, students without knowledge distillation, and students with knowledge distillation with the optimal temperature (T = 10) value over UCF11, HMDB51, UCF50, and UCF101 datasets, respectively. The middle plots in Figure 5 represent the average training losses of the student models trained under the pre-trained 3DCNN teacher model (TFS) using knowledge distillation with different temperature values including 1, 5, 10, 15, and 20. The right-most plots in Figure 5, represent the average training loss of the student model trained under the pre-trained 3DCNN teacher model TUTL using knowledge distillation with different temperature values including 1, 5, 10, 15, and 20. Moreover, the effectiveness of the proposed framework is evaluated with different settings using receiver operating characteristics (ROC) curve and the area under the curve (AUC) values as visualized in Figure 6. Generally, the ROC estimates the contrast between the true positive rate (TPR) and the false positive rate (FPR) for classifier predictions. In Figure 6, the first column represents the ROC curves for the student models trained with and without knowledge distillation under the TFS teacher model with different temperature values over UCF11, HMDB51, UCF50, and UCF101 datasets. The second column in Figure 6 represents the ROC curves for the student models trained with and without knowledge distillation under the TUTL teacher model with different temperature values over UCF11, HMDB51, UCF50, and UCF101 datasets. As it can be perceived from Figure 6, the proposed framework with different settings obtains the best AUC values and ROC curves across all datasets.

We have also compared the accuracy of the proposed framework with different knowledge distillation settings (such as knowledge distillation with two different teacher models including the TFS and TUTL, and different temperature values). The obtained quantitative results comparisons for UCF11, HMDB51, UCF50, and UCF101 are presented in Table 3, Table 4, Table 5 and Table 6, respectively. From the quantitative results given in Table 3, it can be noticed that the proposed framework attains different accuracies with different teachers (i.e., TFS and TUTL) and temperature values. For instance, the proposed 3DCNN student model achieves the best and the runner-up accuracies of 98.78% and 98.17%, respectively, when distilled by the TUTL teacher model having temperature values of T = 10 and T = 15, respectively. Furthermore, it is worth noticing that the proposed distilled 3DCNN model obtains approximately 10% improvement in accuracy in comparison with the proposed 3DCNN student model trained without knowledge distillation, which has an accuracy of 88.71%. Similarly, for the HMDB51 dataset in Table 4, the proposed 3DCNN student model achieves the best accuracy of 92.89% when distilled by TUTL with T = 10 and obtains the runner-up accuracy of 91.55% when distilled by TFS with T = 10. Furthermore, it can be perceived from Table 4 that the proposed TUTL-distilled 3DCNN student model obtains approximately 4.65% improvement in accuracy in comparison with the proposed 3DCNN student model trained without knowledge distillation. For the UCF50 dataset in Table 5, the proposed 3DCNN student model attains the best accuracy of 97.71% when distilled by TUTL with T = 10 and achieves the runner-up accuracy of 97.60% when distilled by TFS with T = 10. Moreover, the proposed 3DCNN student model when distilled by TUTL with T = 10 obtains around 2% improvement in accuracy in comparison with the proposed 3DCNN student model trained without knowledge distillation. Finally, for the UCF101 dataset in Table 6, the proposed 3DCNN student model achieves the best and runner-up accuracies of 97.36% and 96.80%, respectively, when distilled by TUTL with temperature values of T = 10 and T = 15, respectively. Furthermore, the proposed 3DCNN student model attains approximately 3.62% improvement in accuracy in comparison with the proposed 3DCNN student model trained without knowledge distillation. Furthermore, from the reported results in Table 3, Table 4, Table 5 and Table 6, it can be noticed that the proposed 3DCNN student model performs well when trained with the TUTL teacher model having temperature value T = 10 as compared to the proposed TFS-distilled 3DCNN student model with other temperature values. Thus, based on the obtained quantitative results, we determine that the proposed 3DCNN performs well when trained under TUTL with temperature value T = 10 as compared to other settings across all datasets we used in our experiments.

### 4.4. Comparison with the State-of-the-Art Methods

In this section, we compare our proposed framework with the state-of-the-art human action recognition methods with and without knowledge distillation. The detailed quantitative comparative assessment of the proposed framework with non-distillation state-of-the-art human action recognition methods on UCF11, HMDB51, UCF50, and UCF101 datasets are presented in Table 7, Table 8, Table 9 and Table 10, respectively. For instance, for the UCF11 dataset, the proposed Student${}_{3DCNN}$-TUTL method surpasses the state-of-the-art methods by obtaining the best accuracy of 98.7%, whereas the STDAN method [44] attains the second-best accuracy of 98.2%. Amongst all the compared methods, the local-global features + QSVM method [45] has the lowest accuracy of 82.6% for UCF11 dataset whereas the rest of the methods including multi-task hierarchical clustering [46], BT-LSTM [47], deep autoencoder [48], two-stream attention-LSTM [49], weighted entropy-variances based feature selection [50], dilated CNN + BiLSTM-RB [51], and DS-GRU [52] achieve accuracies of 89.7%, 85.3%, 96.2%, 96.9%, 94.5%, 89.0%, and 97.1%, respectively, for the UCF11 dataset.

For the HMDB51 dataset, which is one of the most challenging video dataset, our proposed Student${}_{3DCNN}$-TUTL method achieves the overwhelming results by obtaining an accuracy of 92.8%, whereas the evidential deep learning method [62] achieves runner-up results with an accuracy of 77.0%. The multi-task hierarchical clustering method [46] attains an accuracy of 51.4%, which is the lowest accuracy amongst all comparative methods over the HMDB51 dataset. The other comparative methods, that include STPP + LSTM [53], optical-flow + multi-layer LSTM [54], TSN [55], IP-LSTM [56], deep autoencoder [48], TS-LSTM + temporal-inception [57], HATNet [58], correlational CNN + LSTM [59], STDAN [44], DB-LSTM + SSPF [60], DS-GRU [52], TCLC [61], and semi-supervised temporal gradient learning [63] achieves 70.5%, 72.2%, 70.7%, 58.6%, 70.3%, 69.0%, 74.8%, 66.2%, 56.5%, 75.1%, 72.3%, 71.5%, and 75.9% accuracies, respectively, for the HMDB51 dataset.

For the UCF50 dataset, the proposed Student${}_{3DCNN}$-TUTL method outperforms all the comparative methods by obtaining the best accuracy of 97.6% followed by the runner-up LD-BF + LD-DF method [65], which achieves an accuracy of 97.5%. The local-global features + QSVM [45] method attains the lowest accuracy of 69.4% amongst all comparative method on the UCF50 dataset. The rest of the comparative methods including multi-task hierarchical clustering [46], deep autoencoder [48], ensembled swarm-based optimization [64], and DS-GRU [52] achieve accuracies of 93.2%, 96.4%, 92.2%, and 96.4%, respectively, for the UCF50 dataset.

Finally, for the UCF101 dataset, the proposed Student${}_{3DCNN}$-TUTL method surpasses all the comparative methods by obtaining the best accuracy of 97.3%, followed by the RTS method [74], which attains the second best accuracy of 96.4%. The multi-task hierarchical clustering [46] obtains the lowest accuracy of 76.3% amongst all the comparative methods for the UCF101 dataset. The rest of comparative methods that include saliency-aware 3DCNN with LSTM [66], spatio-temporal multilayer networks [67], long-term temporal convolutions [21], CNN + Bi-LSTM [68], OFF [69], TVNet [70], attention cluster [71], Videolstm [18], two stream convnets [72], mixed 3D-2D convolutional tube [73], TS-LSTM + temporal-inception [57], TSN + TSM [75], STM [76], and correlational CNN + LSTM [59] obtain accuracies of 84.0%, 87.0%, 82.4%, 92.8%, 96.0%, 95.4%, 94.6%, 89.2%, 84.9%, 88.9%, 91.1%, 94.3%, 96.2%, and 92.8%, respectively, for the UCF101 dataset.

To further validate the performance generalization of our method, we compute the confidence interval as in [77] of our proposed method for each dataset used in this paper and compare it with the average confidence interval of the state-of-the-art methods. It is worth mentioning here that we estimate the confidence interval for our method and the state-of-the-art methods using a confidence level of 95%. The obtained confidence interval values of the proposed method and the state-of-the-art methods are listed in Table 11. From the obtained confidence interval values, it can be perceived that the proposed method has the higher confidence with small interval on UCF11 dataset as compare to the state-of-the-art methods. For instance, the proposed method has the confidence interval between 97.59 and 99.96 with a small range of 2.37, where as the state-of-the-art methods have the average confidence interval between 87.81 and 96.52 with a comparatively large range of 8.72. Similarly, for the HMDB51 dataset, the proposed method has the confidence interval between 91.46 and 94.20 with a small range of 2.74, whereas the state-of-the-art methods have the average confidence interval between 64.62 and 72.98 with a comparatively large range of 8.36. For the UCF50 dataset, the proposed method has the confidence interval between 96.78 and 98.48 with a small range of 1.65, whereas the state-of-the-art methods have the average confidence interval between 79.53 and 97.48 with a comparatively large range of 18.98. Finally, for the UCF101 dataset, the proposed methods has the confidence interval between 96.72 and 97.94 with a small range of 1.22, whereas the state-of-the-art methods have the average confidence interval between 87.44 and 93.27 with comparatively large range of 6.27. It is worth noticing that the proposed method has a higher confidence across each dataset with a small interval as compare to the state-of-the-art methods, which verfies the effectiveness of the proposed method over the state-of-the-art methods.

Beside comparing the proposed framework with the mainstream human action recognition methods, we also compare our proposed framework with the knowledge distillation-based human action recognition methods. The comparative analysis of the proposed method with the state-of-the-art knowledge distillation-based human action recognition methods on HMDB51 and UCF101 datasets are presented in Table 12 and Table 13, respectively. The results listed in Table 12 demonstrates the overwhelming performance of the proposed method on HMDB51 dataset in comparison with other knowledge distillation-based methods. For instance, the proposed method achieves the best accuracy of 92.8% amongst all the comparative methods, followed by the runner-up D3D method D3D [32], which obtains an accuracy of 78.7%. The SKD-SRL [33] method attains the lowest accuracy of 29.8% amongst all the comparative knowledge distillation-based methods for the HMDB51 dataset. The rest of comparative methods that include STDDCN [29], Prob-Distill [30], MHSA-KD [31], TY [34], (2+1)D Distilled ShuffleNet [36], and Self-Distillation (PPTK) [35] achieve accuracies of 66.8%, 72.2%, 57.8%, 32.8%, 59.9%, and 76.5%, respectively, for the HMDB51 dataset. Similarly, for the UCF101 dataset in Table 13, our proposed framework outperforms other comparative knowledge distillation-based methods by obtaining the best accuracies of 97.3% followed by the D3D [32] method which attains the second-best accuracy of 97.0%. The TY [34] method achieves the lowest accuracy of 71.1% amongst all the comparative methods for the UCF101 dataset. The rest of comparative methods that include STDDCN [29], Prob-Distill [30], SKD-SRL [33], Progressive KD [37], (2+1)D Distilled ShuffleNet [36], Self-Distillation (PPTK) [35], and ConDi-SR [38] achieve accuracies of 93.7%, 95.7%, 71.9%, 88.8%, 86.4%, 94.6%, and 91.2%, respectively, for the UCF101 dataset.

We also evaluate the performance generalization of our proposed method in comparison with the state-of-the-art knowledge-distillation based methods using confidence interval (with a confidence level of 95%) on HMDB51 and UCF101 datasets. The obtained confidence interval values of the proposed method and the state-of-the-art methods are listed in Table 14. It is clear from the obtained confidence interval values that the proposed method achieves better confidence interval values as compare to the state-of-the-art methods across both HMDB51 and UCF101 datasets. For instance, the proposed method has the confidence interval on the HMDB51 dataset in between 91.46 and 94.20 with a small range of 2.74, whereas the state-of-the-art methods have the average confidence interval between 43.59 and 75.03 with a comparatively very large range of 31.44. Similarly, for the UCF101 dataset, the proposed method has the confidence interval between 96.72 and 97.94 with a small range of 1.22, whereas the state-of-the-art methods have the average confidence interval between 80.29 and 95.34 with a comparatively large range of 15.11. The obtained confidence interval values for both the datasets verify the generalization and effectiveness of the proposed method over the state-of-the-art knowledge distillation-based methods.

Considering the overall comparative analysis across all datasets, the proposed framework outperforms all the comparative mainstream action recognition methods by obtaining an improvement in accuracy of 7%, 34.88%, 7.7%, and 8% on UCF11, HMDB51, UCF50, and UCF101 datasets, respectively. Furthermore, we compare our proposed framework with knowledge distillation-based human action recognition methods on HMDB51 and UCF101 datasets. Experimental results reveal that our proposed framework attains an improvement in accuracy of 56.46% and 6.39%, on average, on HMDB51 and UCF101 datasets, respectively, over the existing knowledge distillation-based human action recognition methods.

### 4.5. Run Time Analysis

To validate the effectiveness and suitability of the proposed methods for practical applications in real-time environment, we have computed the inference time of the proposed method for action recognition task in terms of seconds per frame (SPF) and frames per second (FPS) with and without GPU computational resources. The obtained run time results are then compared with the state-of-the-art human actions recognition methods and presented in Table 15. The run time results listed in Table 15 shows that, while using GPU resources OFF [69] has the best inference time results with the SPF of 0.0048 and FPS of 206, followed by STPP + LSTM [53] having the second-best run time results with the SPF of 0.0053 and FPS of 186.6. The proposed framework attains the third best run time results with the SPF of 0.0091 and FPS of 110. The Videolstm [18] method has the worst run time results with SPF of 0.0940 and FPS of 10.6 among all comparative methods while using GPU resources. When using CPU resources, the propose method obtains the best run time results with the SPF of 0.0106 and FPS of 93. The Optical-flow + multi-layer LSTM [54] has the second-best run time results with the SPF of 0.18 and FPS of 3.5.

To provide a fair comparison of the inference speed, we scaled [78] the run time results of the state-of-the-art methods in Table 15 to the hardware specifications (i.e., 2.5 GHz CPU and 585 MHz GPU) we used in our experiments. The scaled run time results of the proposed method and other comparative human action recognition methods are presented in Table 16. From the scaled results in Table 16, it can be noticed that the STPP + LSTM [53] method has the best SPF and FPS values of 0.0063 and 154.6, respectively, for the GPU inference. The OFF [69] method has the second-best SPF and FPS values of 0.0082 and 120.5, respectively, followed by the proposed method having the third best SPF and FPS values of 0.0091 and 110, respectively, for the GPU inference. On the other hand, for inference on the CPU, our proposed method attains the best SPF and FPS of 0.0106 and 93, respectively, followed by Optical-flow + multi-layer LSTM [54], which has the runner-up SPF and FPS values of 0.23 and 2.6, respectively. It is worth mentioning here that, the proposed method provides comparatively slower inference speed than STPP + LSTM [53] and OFF [69] on GPU resources, however, the proposed method is more efficient and robust in terms of accuracy as compare to STPP + LSTM [53] and OFF [69]. Moreover, for CPU inference, the proposed method outperforms the comparative methods by obtaining the best SPF and FPS values for both scaled and unscaled inference speed analysis by obtaining an improvement of up to 28× and 37× for SPF metric and an improvement of 37× and 50× for FPS metric, respectively. It is also worth mentioning here that the proposed framework not only obtains the best accuracy on the UCF11 dataset but also attains an improvement up to 2× in terms of FPS metric over the runner-up STDAN [44] method. Similarly, on the UCF50 dataset, the proposed method obtains an improvement of up to 13× in terms of FPS metric over the runner-up LD-BF + LD-DF [65] method. Thus, the obtained quantitative and run time assessment results validate the efficiency and robustness of our proposed framework for real-time human action recognition task.

## 5. Conclusions

In this work, we have proposed a knowledge distillation-driven 3DCNN framework for vision-based human action recognition task. The proposed framework uses offline knowledge distillation approach to transfer the spatio-temporal knowledge from a large 3DCNN teacher model to a lightweight 3DCNN model. During the offline knowledge distillation training, the 3DCNN student model learns from predictions of the pre-trained 3DCNN teacher model and gradually improves it predictions performance. Using the offline knowledge distillation approach, we not only transfer the knowledge but also perform model compression, thereby, transferring the knowledge from a large teacher model to a small student mode, which offers a similar level of prediction accuracy as the teacher model for the human action recognition task. To evaluate the performance of the proposed framework, we have conducted extensive experiments with different settings on four benchmark human action recognition datasets that include UCF11, HMDB51, UCF50, and UCF101. We have compared our proposed framework with the mainstream human activity recognition methods as well as knowledge distillation-based human action recognition methods. The obtained experimental and comparative analysis validate the effectiveness of our proposed framework over state-of-the-art human action recognition methods. Experimental results show that our proposed framework obtains an accuracy improvement of 7%, 34.88%, 7.7%, and 8%, on average, for UCF11, HMDB51, UCF50, and UCF101 datasets, respectively, as compared to the state-of-the-art human action recognition methods. To further validate the performance generalization of our method, we have computed the confidence interval of our proposed method for each dataset used in this paper and compared it with the average confidence interval of the state-of-the-art methods. Our obtained confidence interval results indicate that our proposed method attains a higher confidence with small intervals as compare to the state-of-the-art methods. Besides, we have analyzed the run time performance of the proposed framework with other comparative methods in terms of SPF and FPS metrics for both GPU and CPU execution environments. The comparative results demonstrate that the proposed framework achieves a comparable inference speed on GPU while obtaining better accuracy whereas the proposed framework obtains a run time improvement of up to 37× in terms of SPF and 50× in terms of FPS on CPU over existing methods while also attaining better accuracy than existing methods. These experimental results demonstrate the suitability of our proposed framework for human action recognition task in real-time environments.

The current version of our proposed framework uses plain 3DCNN teacher and student models with offline knowledge distillation mechanism. The obtained results validate the effectiveness of the current version of the proposed framework; however, in our future work, we plan to analyze residual 3DCNN and self-distillation instead of offline distillation.

## Figures and Tables

**Table 1:** Architectural details of the proposed 3DCNN teacher Model.

Layer	Input Channels	Number of Kernels	Kernel Size	Activation	Padding	Output Channels
Conv 1	3	64	3×3×3	ReLU	1	64
3D Maxpooling Layer						
Conv 2	64	128	3×3×3	ReLU	1	128
3D Maxpooling Layer						
Conv 3a	128	256	3×3×3	ReLU	1	256
Conv 3b	256	256	3×3×3	ReLU	1	256
3D Maxpooling Layer						
Conv 4a	256	512	3×3×3	ReLU	1	512
Conv 4b	512	512	3×3×3	ReLU	1	512
3D Maxpooling Layer						
Conv 5a	512	512	3×3×3	ReLU	1	512
Conv 5b	512	512	3×3×3	ReLU	1	512
3D Maxpooling Layer						
FC1-(4096)						
FC2-(4096)						
Softmax (Number of classes)						

**Table 2:** Architectural details of the proposed 3DCNN student Model.

Layer	Input Channels	Number of Kernels	Kernel Size	Activation	Padding	Output Channels
Conv 1	3	16	3×3×3	ReLU	1	16
3D Maxpooling Layer						
Conv 2	16	32	3×3×3	ReLU	1	32
3D Maxpooling Layer						
Conv 3a	32	32	3×3×3	ReLU	1	32
Conv 3b	32	32	3×3×3	ReLU	1	32
3D Maxpooling Layer						
Conv 4a	32	64	3×3×3	ReLU	1	64
Conv 4b	64	64	3×3×3	ReLU	1	64
3D Maxpooling Layer						
Conv 5a	64	128	3×3×3	ReLU	1	128
Conv 5b	128	128	3×3×3	ReLU	1	128
3D Maxpooling Layer						
FC1-(2048)						
FC2-(1024)						
Softmax (Number of classes)						

**Table 3:** Quantitative comparative analysis of the results obtained by our proposed framework with and without knowledge distillation (KD) with different settings over UCF11 dataset.

Model	Dataset	Accuracy (%)
Student without KD	UCF11	88.71
TFS Teacher	UCF11	95.86
TUTL Teacher	UCF11	99.70
Student${}_{3DCNN}$-TFS (T = 1)	UCF11	95.73
Student${}_{3DCNN}$-TFS (T = 15)	UCF11	96.84
Student${}_{3DCNN}$-TFS (T = 10)	UCF11	97.56
Student${}_{3DCNN}$-TFS (T = 15)	UCF11	96.43
Student${}_{3DCNN}$-TFS (T = 20)	UCF11	96.58
Student${}_{3DCNN}$-TUTL (T = 1)	UCF11	95.12
Student${}_{3DCNN}$-TUTL (T = 15)	UCF11	97.45
Student${}_{3DCNN}$-TUTL (T = 10)	UCF11	**98.78**
Student${}_{3DCNN}$-TUTL (T = 15)	UCF11	*98.17*
Student${}_{3DCNN}$-TUTL (T = 20)	UCF11	97.88

The best and runner-up accuracy scores are highlighted in bold and italic text, respectively.

**Table 4:** Quantitative comparative analysis of the results obtained by our proposed framework with and without knowledge distillation (KD) with different settings over HMDB51 dataset.

Model	Dataset	Accuracy (%)
Student without KD	HMDB51	88.24
TFS Teacher	HMDB51	90.67
TUTL Teacher	HMDB51	93.10
Student${}_{3DCNN}$-TFS (T = 1)	HMDB51	88.62
Student${}_{3DCNN}$-TFS (T = 15)	HMDB51	88.25
Student${}_{3DCNN}$-TFS (T = 10)	HMDB51	*91.55*
Student${}_{3DCNN}$-TFS (T = 15)	HMDB51	87.22
Student${}_{3DCNN}$-TFS (T = 20)	HMDB51	86.92
Student${}_{3DCNN}$-TUTL (T = 1)	HMDB51	89.80
Student${}_{3DCNN}$-TUTL (T = 15)	HMDB51	89.14
Student${}_{3DCNN}$-TUTL (T = 10)	HMDB51	**92.89**
Student${}_{3DCNN}$-TUTL (T = 15)	HMDB51	89.43
Student${}_{3DCNN}$-TUTL (T = 20)	HMDB51	89.66

The best and runner-up accuracy scores are highlighted in bold and italic text, respectively.

**Table 5:** Quantitative comparative analysis of the results obtained by our proposed framework with and without knowledge distillation (KD) with different settings over UCF50 dataset.

Model	Dataset	Accuracy (%)
Student without KD	UCF50	95.81
TFS Teacher	UCF50	96.40
TUTL Teacher	UCF50	98.37
Student${}_{3DCNN}$-TFS (T = 1)	UCF50	96.25
Student${}_{3DCNN}$-TFS (T = 15)	UCF50	96.70
Student${}_{3DCNN}$-TFS (T = 10)	UCF50	*97.60*
Student${}_{3DCNN}$-TFS (T = 15)	UCF50	96.91
Student${}_{3DCNN}$-TFS (T = 20)	UCF50	97.21
Student${}_{3DCNN}$-TUTL (T = 1)	UCF50	96.65
Student${}_{3DCNN}$-TUTL (T = 15)	UCF50	97.15
Student${}_{3DCNN}$-TUTL (T = 10)	UCF50	**97.71**
Student${}_{3DCNN}$-TUTL (T = 15)	UCF50	97.53
Student${}_{3DCNN}$-TUTL (T = 20)	UCF50	97.64

The best and runner-up accuracy scores are highlighted in bold and italic text, respectively.

**Table 6:** Quantitative comparative analysis of the results obtained by our proposed framework with and without knowledge distillation (KD) with different settings over UCF101 dataset.

Model	Dataset	Accuracy (%)
Student without KD	UCF101	93.74
TFS Teacher	UCF101	95.04
TUTL Teacher	UCF101	98.83
Student${}_{3DCNN}$-TFS (T = 1)	UCF101	95.11
Student${}_{3DCNN}$-TFS (T = 15)	UCF101	95.64
Student${}_{3DCNN}$-TFS (T = 10)	UCF101	96.73
Student${}_{3DCNN}$-TFS (T = 15)	UCF101	96.11
Student${}_{3DCNN}$-TFS (T = 20)	UCF101	96.20
Student${}_{3DCNN}$-TUTL (T = 1)	UCF101	96.24
Student${}_{3DCNN}$-TUTL (T = 15)	UCF101	*96.80*
Student${}_{3DCNN}$-TUTL (T = 10)	UCF101	**97.36**
Student${}_{3DCNN}$-TUTL (T = 15)	UCF101	96.58
Student${}_{3DCNN}$-TUTL (T = 20)	UCF101	95.90

The best and runner-up accuracy scores are highlighted in bold and italic text, respectively.

**Table 7:** Quantitative comparative analysis of our proposed framework with the state-of-the-art action recognition methods on UCF11 dataset.

Model	Year	Accuracy (%)
Multi-task hierarchical clustering [46]	2016	89.7
BT-LSTM [47]	2018	85.3
Deep autoencoder [48]	2019	96.2
STDAN [44]	2020	*98.2*
Two-stream Attention-LSTM [49]	2020	96.9
Weighted entropy-variances based feature selection [50]	2021	94.5
Dilated CNN + BiLSTM-RB [51]	2021	89.0
DS-GRU [52]	2021	97.1
Local-global features + QSVM [45]	2021	82.6
Student${}_{3DCNN}$-TUTL (Ours)	2023	**98.7**

The best and runner-up accuracy scores are highlighted in bold and italic text, respectively.

**Table 8:** Quantitative comparative analysis of our proposed method with the state-of-the-art action recognition methods on HMDB51 dataset.

Model	Year	Accuracy (%)
Multi-task hierarchical clustering [46]	2016	51.4
STPP + LSTM [53]	2017	70.5
Optical-flow + Multi-layer LSTM [54]	2018	72.2
TSN [55]	2018	70.7
IP-LSTM [56]	2019	58.6
Deep autoencoder [48]	2019	70.3
TS-LSTM + Temporal-inception [57]	2019	69.0
HATNet [58]	2019	74.8
Correlational CNN + LSTM [59]	2020	66.2
STDAN [44]	2020	56.5
DB-LSTM + SSPF [60]	2021	75.1
DS-GRU [52]	2021	72.3
TCLC [61]	2021	71.5
Evidential deep learning [62]	2021	*77.0*
Semi-supervised temporal gradient learning [63]	2022	75.9
Student${}_{3DCNN}$-TUTL (Ours)	2023	**92.8**

The best and runner-up accuracy scores are highlighted in bold and italic text, respectively.

**Table 9:** Quantitative comparative analysis of our proposed method with the state-of-the-art action recognition methods on UCF50 dataset.

Model	Year	Accuracy (%)
Multi-task hierarchical clustering [46]	2016	93.2
Deep autoencoder [48]	2019	96.4
Ensembled swarm-based optimization [64]	2021	92.2
DS-GRU [52]	2021	95.2
Local-global features + QSVM [45]	2021	69.4
LD-BF + LD-DF [65]	2022	*97.5*
Student${}_{3DCNN}$-TUTL (Ours)	2023	**97.6**

The best and runner-up accuracy scores are highlighted in bold and italic text, respectively.

**Table 10:** Quantitative comparative analysis of our proposed method with the state-of-the-art action recognition methods on UCF101 dataset.

Model	Year	Accuracy (%)
Multi-task hierarchical clustering [46]	2016	76.3
Saliency-aware 3DCNN with LSTM [66]	2016	84.0
Spatio-temporal multilayer networks [67]	2017	87.0
Long-term temporal convolutions [21]	2017	82.4
CNN + Bi-LSTM [68]	2017	92.8
OFF [69]	2018	96.0
TVNet [70]	2018	95.4
Attention cluster [71]	2018	94.6
Videolstm [18]	2018	89.2
Two stream convnets [72]	2018	84.9
Mixed 3D-2D convolutional tube [73]	2018	88.9
RTS [74]	2019	*96.4*
TS-LSTM + Temporal-inception [57]	2019	91.1
TSN + TSM [75]	2019	94.3
STM [76]	2019	96.2
Correlational CNN + LSTM [59]	2020	92.8
Student${}_{3DCNN}$-TUTL (Ours)	2023	**97.3**

The best and runner-up accuracy scores are highlighted in bold and italic text, respectively.

**Table 11:** Computed confidence interval values (with 95% confidence) for our proposed method and the state-of-the-art mainstream methods.

Dataset	State-of-the-Art Methods	Student${}_{3\mathrm{DCNN}}$-TUTL (Ours)
UCF11	[87.81–96.52]	[97.59–99.96]
HMDB51	[64.62–72.98]	[91.46–94.20]
UCF50	[79.53–97.48]	[96.78–98.42]
UCF101	[87.44–93.27]	[96.72–97.94]

First value in the square brackets represents the lower bound and the second value represents the upper bound. Together the lower and the upper bound represent the confidence interval.

**Table 12:** Quantitative comparative analysis of our proposed method with the state-of-the-art knowledge distillation-based action recognition methods on HMDB51 dataset.

Model	Year	Accuracy (%)
STDDCN [29]	2019	66.8
Prob-Distill [30]	2019	72.2
MHSA-KD [31]	2019	57.8
D3D [32]	2020	*78.7*
SKD-SRL [33]	2021	29.8
TY [34]	2021	32.8
(2+1)D Distilled ShuffleNet [36]	2022	59.9
Self-Distillation (PPTK) [35]	2022	76.5
Student${}_{3DCNN}$-TUTL (Ours)	2023	**92.8**

The best and runner-up accuracy scores are highlighted in bold and italic text, respectively.

**Table 13:** Quantitative comparative analysis of our proposed method with the state-of-the-art knowledge distillation-based action recognition methods on UCF101 dataset.

Model	Year	Accuracy (%)
STDDCN [29]	2019	93.7
Prob-Distill [30]	2019	95.7
D3D [32]	2020	*97.0*
SKD-SRL [33]	2021	71.9
Progressive KD [37]	2021	88.8
TY [34]	2021	71.1
(2+1)D Distilled ShuffleNet [36]	2022	86.4
Self-Distillation (PPTK) [35]	2022	94.6
ConDi-SR [38]	2022	91.2
Student${}_{3DCNN}$-TUTL (Ours)	2023	**97.3**

The best and runner-up accuracy scores are highlighted in bold and italic text, respectively.

**Table 14:** Computed confidence interval values (with 95% confidence) for our proposed method and the state-of-the-art knowledge distillation-based methods.

Dataset	State-of-the-Art Methods	Student${}_{3\mathrm{DCNN}}$-TUTL (Ours)
HMDB51	[43.59–75.03]	[91.46–94.20]
UCF101	[80.29–95.34]	[96.72–97.94]

First value in the square brackets represents the lower bound and the second value represents the upper bound. Together the lower and upper bound represent the confidence interval.

**Table 15:** Run time analysis of our proposed framework with the state-of-the-art human action recognition methods (without scaling).

Method	Seconds per Frame (SPF)		Year	Frames per Second (FPS)	
	GPU	CPU		GPU	CPU
STPP + LSTM [53]	*0.0053*	-	2017	*186.6*	-
CNN + Bi-LSTM [68]	0.0570	-	2017	20	-
OFF [69]	**0.0048**	-	2018	**206**	-
Videolstm [18]	0.0940	-	2018	10.6	-
Optical-flow + Multi-layer LSTM [54]	0.0356	*0.18*	2018	30	*3.5*
Deep autoencoder [48]	0.0430	0.43	2019	24	1.5
TSN + TSM [75]	0.0167	-	2019	60	-
IP-LSTM [56]	0.0431	-	2019	23.2	-
STDAN [44]	0.0075	-	2020	132	-
DS-GRU [52]	0.0400	-	2021	25	-
LD-BF + LD-DF [65]	0.0670	-	2022	14	-
Student${}_{3DCNN}$-TUTL (Ours)	0.0091	**0.0106**	2023	110	**93**

The best and runner-up SPF and FPS scores for GPU and CPU are highlighted in bold and italic text, respectively.

**Table 16:** Run time analysis of our proposed framework with the state-of-the-art human action recognition methods (with scaling).

Method	Seconds per Frame (SPF)		Year	Frames per Second (FPS)	
	GPU	CPU		GPU	CPU
STPP + LSTM [53]	**0.0063**	-	2017	**154.6**	-
CNN + Bi-LSTM [68]	0.0974	-	2017	11.7	-
OFF [69]	*0.0082*	-	2018	*120.5*	-
Videolstm [18]	0.1606	-	2018	6.2	-
Optical-flow + Multi-layer LSTM [54]	0.0608	*0.23*	2018	17.5	*2.6*
Deep autoencoder [48]	0.0735	0.56	2019	14	1.1
TSN + TSM [75]	0.0458	-	2019	21.8	-
IP-LSTM [56]	0.0736	-	2019	13.57	-
STDAN [44]	0.0128	-	2020	77.2	-
DS-GRU [52]	0.0683	-	2021	14.6	-
LD-BF + LD-DF [65]	0.1145	-	2022	8.1	-
Student${}_{3DCNN}$-TUTL (Ours)	0.0091	**0.0106**	2023	110	**93**

The best and runner-up SPF and FPS scores for graphics processing unit (GPU) and central processing unit (CPU) are highlighted in bold and italic text, respectively.

## Data Availability

The datasets generated during and/or analysed during the current study are available from the corresponding author on reasonable request.

## Abbreviations

DNNs	Deep neural networks
CNN	Convolutional neural network
3DCNN	3D Convolutional neural network
ROC	Receiver operation characteristics
AUC	Area under the curve
SPF	Seconds per frame
FPS	Frames per seocnd
TFS	Train from scratch
TUTL	Train using transfer learning
